# Supplementary material for: Elevated developmental temperatures below the lethal limit reduce Aedes aegypti fertility
Source: J Exp Biol. 2025 Feb 7;228(3):JEB249803. doi: 10.1242/jeb.249803 (PMC11832123; doi:10.1242/jeb.249803)
Supplement: Supplementary information [file jexbio-228-249803-s1.pdf]

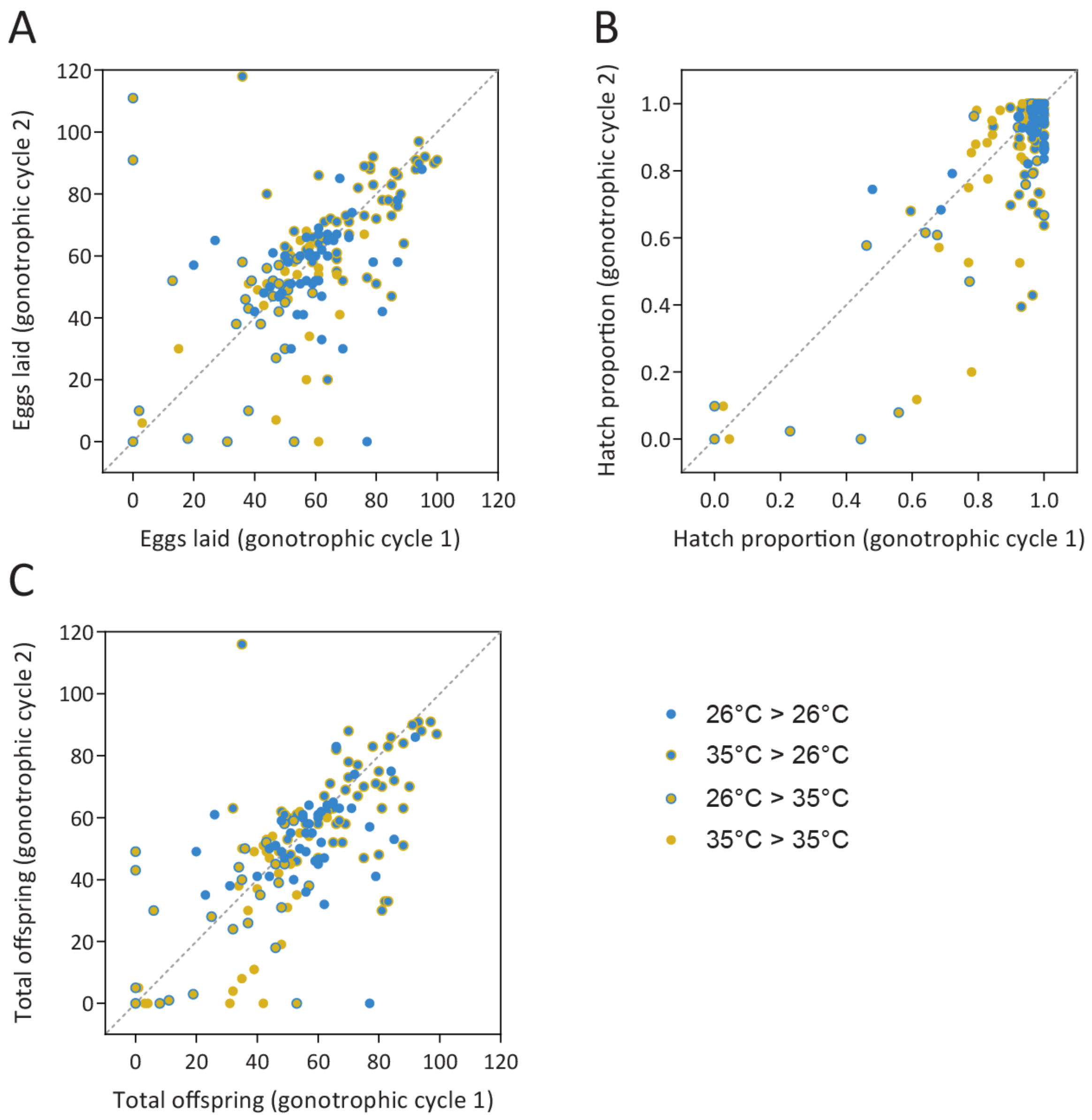

Fig. S1. Correlations in (A) fecundity, (B) egg hatch proportions and (C) total offspring between the first and second gonotrophic cycles. Dots represent data from individual females measured across two gonotrophic cycles in the experiment testing cross-generational effects of elevated developmental temperatures also shown in Fig. 7.

Table S1. Analyses of fecundity and egg hatch proportions under a zero inflation model with zero counts inlcuded.

Response: Fecundity	Chisq	Df	Pr(>Chisq)
Female temperature	66.8548	1	2.923e-16 ***
Male temperature	4.0866	1	0.04322 *
Female temperature*Male temperature	0.4246	1	0.51463
Response: Egg hatch proportion	Chisq	Df	Pr(>Chisq)
Female temperature	0.0024	1	0.960976
Male temperature	6.7906	1	0.009164 **
Female temperature*Male temperature	0.407	1	0.523491
